# Quantum pattern recognition algorithms for charged particle tracking

**DOI:** 10.1098/rsta.2021.0103

**Published:** 2022-02-07

**Authors:** H. M. Gray

**Affiliations:** ^1^ Physics Department, University of California, Berkeley, CA 947200, USA; ^2^ Physics Division, Lawrence Berkeley Laboratory, 1 Cyclotron Road, Berkeley, CA 94720, USA

**Keywords:** quantum computing, track reconstruction, pattern recognition, quantum machine learning

## Abstract

High-energy physics is facing a daunting computing challenge with the large datasets expected from the upcoming High-Luminosity Large Hadron Collider in the next decade and even more so at future colliders. A key challenge in the reconstruction of events of simulated data and collision data is the pattern recognition algorithms used to determine the trajectories of charged particles. The field of quantum computing shows promise for transformative capabilities and is going through a cycle of rapid development and hence might provide a solution to this challenge. This article reviews current studies of quantum computers for charged particle pattern recognition in high-energy physics.

This article is part of the theme issue ‘Quantum technologies in particle physics’.

## 1. Introduction

The high-energy physics experiments at the Large Hadron Collider (LHC) located just outside Geneva, Switzerland have transformed the field of particle physics, most notably through the exciting discovery of the Higgs boson [1,2] by the ATLAS [3] and CMS [4] experiments. While analysis of the data from the LHC is still ongoing, preparations are underway for the daunting computing challenge of the large datasets expected from the upgrade of the LHC, the High-Luminosity LHC (HL-LHC) in the next decade and future colliders to follow the HL-LHC. The physics reach of the HL-LHC relies on an order-of-magnitude higher luminosity than the LHC, however, which means that the number of additional proton-proton interactions that occur each time the beams collide, also referred to as pile up, μ, will increase by a factor of 3–5, reaching values of 140–200 additional interactions per collision. As a result, the computing environment at the HL-LHC will be exceedingly challenging and current projections show that the computing resources that will be required to process the data exceed budget projections.

Pattern recognition algorithms for the reconstruction of the trajectories of charged particles are a key challenge in the reconstruction of events of simulated data and collision data. Pattern recognition algorithms [5] can be broadly categorized as either global or local methods. Global pattern recognition methods find trajectories by processing all measurements from the full detector simultaneously. Examples of global methods include conformal mapping or transform methods, such as the Hough transform [6,7] and neural networks [8]. Local pattern recognition methods generate track seeds from measurements in a localized detector region and then search for additional hits to complete the track candidates. Examples of local methods include the track road and track following methods such as the Kalman filter [9–11]. Pattern recognition algorithms are typically run within a track reconstruction sequence after seed finding. Once the set of deposited energy has been identified by the pattern recognition algorithms, the parameters of the trajectories are determined by fitting algorithms. The parameters used to describe the trajectories depend on the detector geometry, but typically five (or six, if timing information is included) are used. Track parameters typically include the momentum, which is inversely proportional to the curvature, angles describing the direction of propagation and impact parameters to characterize the points of origin.

**Figure 1.**
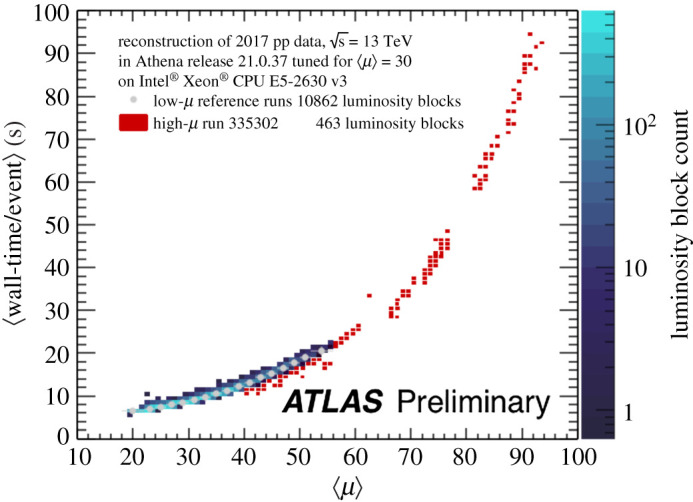
The reconstruction time per event of pattern recognition algorithms in data as a function of the number of additional proton–proton interactions, μ, for the ATLAS detector. The HL-LHC is expected to have μ between 140 and 200, which would lie to the right of the distribution. From [12]. (Online version in colour.)

To illustrate the challenge that will be posed by the HL-LHC, figure 1 shows the dependence of the processing time per event as a function of pile up using data recorded by the ATLAS experiment using a pattern recognition sequence based on the Kalman filter. The processing time scales combinatorially with increasing μ, which is typical of pattern recognition algorithms. At the HL-LHC, the expected value of μ would lie significantly to the right of the curve and hence have large CPU resource requirements. At future hadron colliders, such as the proposed hadron-hadron collider as part of the Future Circular Collider project [13], even more pile up is expected, with potentially up to 1000 additional interactions per event. Due to this challenge, the development of new algorithms and new techniques for pattern recognition for high-energy physics is currently a very active field of development. This article provides an overview of ongoing studies to determine how quantum computers could be used in the future for pattern recognition algorithms.

Quantum computers were first proposed more than 40 years ago [14–16] with initial ideas focused on developing a computer using quantum processes from nature in order to better simulate nature. Further interest was stimulated a decade later through the development of quantum algorithms that demonstrated the potential of quantum computers to solve classically intractable problems, including prime number factorization [17] and search algorithms [18,19]. The first quantum computers were based on existing techniques from nuclear magnetic resonance [20–22]. Recently, we have entered the so-called noisy intermediate-scale quantum (NISQ) era [23] with quantum computers with tens of logical qubits that can surpass the capabilities of current classical computers, albeit limited by significant noise. Qubits are the quantum analogue of the bits used to store information on classical computers.

The quantum computers available today can be categorized as either quantum annealers or circuit-based quantum computers. Quantum annealers are designed to solve a particular type of problem: minimization of objective function and quantum annealing can be expected to solve minimization problems more quickly due to quantum tunnelling. D-Wave produces commercially available quantum annealers with up to 5000 qubits currently available [24].

Circuit-based quantum computers can be used to solve a wider range of problems and, hence, are more similar in concept to the digital computers of today. They consist of quantum circuits made from qubits using a wide range of technologies. Technologies currently being explored for qubits include superconducting transmons, ion traps and topological qubits. IBM, for example, has produced a series of digital quantum computers using superconducting transmon qubits with up to 65 qubits [25].

This article reviews current studies of the potential of quantum computers for charged particle pattern recognition. In many cases, the studies use the open dataset produced in the context of the tracking machine learning challenge (TrackML) [26,27].

## 2. Pattern recognition with quantum annealers

Pattern recognition algorithms for quantum annealers must be formulated as minimization problems. Bapst *et al*. [28] formulate pattern recognition as a quadratic unconstrained binary optimization (QUBO) problem and then determine the global minimum using the D-Wave quantum annealer. The algorithm is a hybrid classical-quantum algorithm with pre- and post-processing performed on the classical computer and the minimization of the QUBO on the quantum annealer.

**Figure 2.**
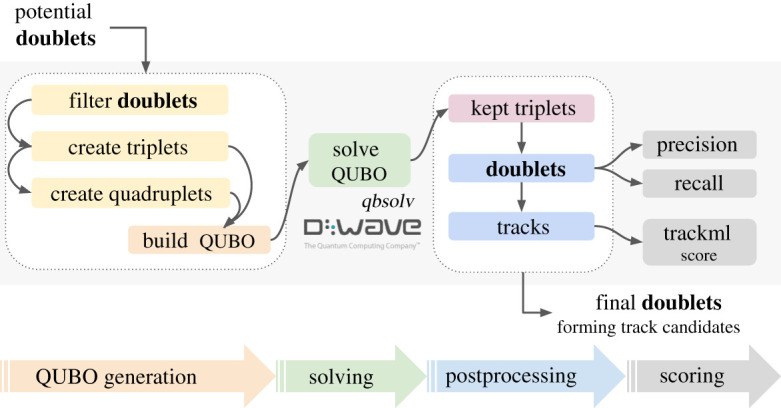
Overview of the steps in the pattern recognition algorithm for quantum annealers. From [28]. (Online version in colour.)

The steps of the quantum pattern recognition algorithm are shown in figure 2. Firstly, the hits corresponding to the energy deposited by particles in the detector are grouped into doublets and then triplets and finally quadruplets. A QUBO is then constructed and configured to identify the best pairs of triplets that can be combined to form a quadruplet. The weights for the objective function are defined based on the compatibility of the parameters of the triplets. In particular, they depend on the compatibility of the curvature of the triplets and the difference in the polar angle between the doublets in the triplet.

The TrackML dataset is used, but simplified by only considering the central part, or barrel region, of the detector, where the modules are oriented parallel to the direction of the beam. In order to study the dependence of the algorithm on pile up, the dataset is filtered by selecting the hits from a random subset of particles and a corresponding fraction of the noise. The software used for the solution of the QUBO is *qbsolv* [29], a tool developed by D-Wave. It uses an iterative hybrid quantum and classical approach and in each iteration, the QUBO is split into smaller sub-QUBOs which are solved for global optimization. This means that the quantum annealers available today can be used to solve QUBO problems that would ideally require quantum annealers with many more qubits. The results of the sub-QUBOs are then combined and a *tabu* search [30] is used for local optimization. The sub-QUBO solving step is run either on a classical computer or on D-Wave.

The simulations were run at the National Energy Research Scientific Computing (NERSC) centre using the Cori supercomputer. Two quantum annealers were used: the Ising D-Wave 2X machine at the Los Alamos National Laboratory and the D-Wave LEAP cloud service. After solving, a post-processing step converts the triplets back into doublets, and any duplicates or doublets with unresolved conflicts are removed. Finally, track candidates with at least five hits are reconstructed from the accepted doublets.

**Figure 3.**
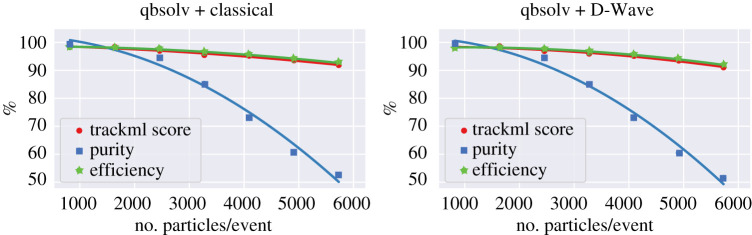
The performance of classical quantum annealing pattern recognition algorithms using a simulator (top) and D-Wave (bottom), as measured by TrackML score (red circle), purity (blue rectangle) and efficiency (green star), as a function of particle multiplicity. From [28]. (Online version in colour.)

Figure 3 shows the performance of this pattern recognition algorithm on classical computers (left) and quantum annealers (right) for events with up to 6000 charged particles. The efficiency is defined as the fraction of true charged particles that are reconstructed and the purity as the fraction of reconstructed tracks that are matched to true particles. The TrackML score is the metric used in the TrackML challenge, which combines efficiency and purity and weights more significantly the performance of particles most important for physics analysis. The efficiency is stable even in events with high multiplicity, however, the purity drops to 50% in the high multiplicity events. In addition, the performance is shown to be the same between the quantum annealers and the classical computers. This demonstrates that the algorithm is robust against noise on the quantum annealers, which could be expected to induce differences between results from the simulations and from the quantum annealing hardware. No detailed timing studies were presented because the quantum annealers used are shared, accessed remotely and inherently stochastic.

**Figure 4.**
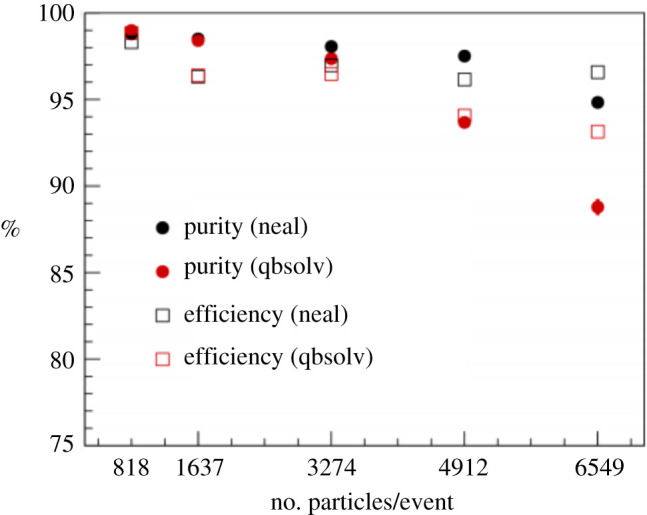
The purity (circles) and efficiency (squares) of the quantum annealing pattern recognition algorithm as a function of the number of charged particles per event. The results from the qbsolv solver are shown in red and the results from the neal solver are shown in black. From [31]. (Online version in colour.)

This pattern recognition algorithm for quantum annealers was extended in [31] by adding information about the track impact parameters into the penalty term of the objective function. Such a criteria is designed to prioritize prompt tracks from the primary interaction and it dramatically improves the purity particularly at events with many particles as shown in figure 4. In addition, the performance using two different software solvers for the QUBO is compared and *neal*, an alternative QUBO solver for simulation from D-Wave, is shown to have better performance than *qbsolv*.

Reference [31] also studied the performance of the annealing algorithms using a Fujitsu digital annealer [32]. This is a specialized annealing device designed to solve QUBO problems using classical simulation of annealing with logic circuits on a custom chip. The digital annealer operates at room temperature. The physics performance of the algorithm was comparable across the different devices, however significant differences in the computation time were observed. Table 1 compares the computing time between the digital annealer and the *neal* solver simulating quantum annealing. The preprocessing and postprocessing time is excluded because it is identical in both algorithms. The time is shown as a function of the density of the event, which is the percentage of charge particles with respect to the expected multiplicity for the HL-LHC. The computing time for the annealer is much larger than that of the CPU, however, the annealing time is essentially independent of the number of tracks and remains below a second and superior to the current performance on quantum annealers for track densities 10% and larger.

**Table 1.** A comparison of the computing time using the digital annealer (DA) and the neal solver. The number of times the dataset is sliced in η is indicated with $N_{\text{slice}}$. The times spent in the queue or with the network for the digital annealer are not included. From [31].

		DA (s)		neal (s)
density [%]	$N_{\text{slice}}$	CPU time	anneal time	total time
5	46	0.09	0.29	0.27
10	68	0.15	0.42	0.66
20	71	0.22	0.44	1.29
40	74	0.52	0.45	2.46
60	73	0.94	0.45	4.29
80	74	1.79	0.46	7.49
100	74	3.73	0.45	12.87

A similar approach to charged particle pattern recognition on quantum annealers is pursued in [33]. The Denby–Peterson method is adapted to perform pattern recognition by classifying track segments. The QUBO is constructed by combining hit doublets into triplets. The terms in the objective function depend on the angles separating the tracks and include a bias term to favour high momentum tracks. The algorithm also exploits the fact that tracks are expected to have originated from the beam spot. Penalties are added for bifurcated tracks in which the hits are not aligned along a potential trajectory and for edges that are not aligned appropriately in the plane parallel to the beam direction.

The TrackML dataset is preprocessed by dividing the event into 32 overlapping sectors in the plane perpendicular to the beam. Hits in the barrel and the endcap of the detector are considered. Candidate edges are selected using Gaussian kernel density estimation to determine the probability that an edge between a pair of hits is true and then the QUBO is divided into smaller optimization problems. The annealing is performed using the D-Wave 2X system.

**Figure 5.**
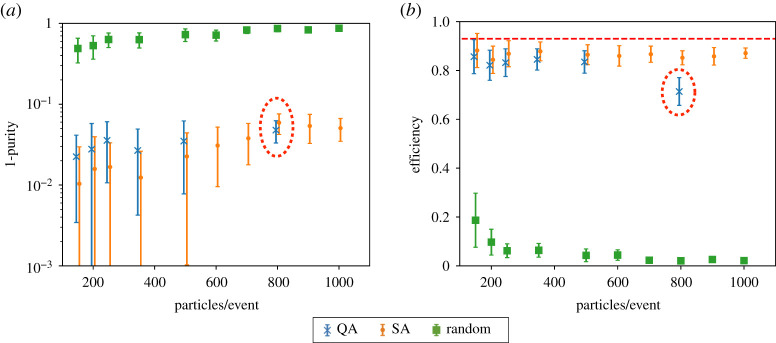
The purity (*a*) and efficiency (*b*) of the quantum annealing (QA, blue), simulated annealing (SA, orange) and random (green) pattern recognition algorithms as a function of the number of particles per event. From [33]. (Online version in colour.)

Figure 5 shows the purity (*a*) and efficiency (*b*) as a function of the number of particles per event. The performance using simulated annealing (SA) and quantum annealing (QA) is compared to the results from a random algorithm. The results for simulation and quantum annealing are similar and the efficiency is typically 90% and the purity above 95%.

## 3. Pattern recognition with associative memory

A different approach to pattern recognition uses associative memory to store the patterns of hits in the detector corresponding to all possible track candidates. When processing data, the pattern recognition algorithms are replaced by retrieving patterns from the associative memory. Such algorithms do not scale combinatorially with the number of hits. Pattern recognition with associative memory has been explored in the context of the development of hardware triggers for fast track reconstruction [34].

**Figure 6.**
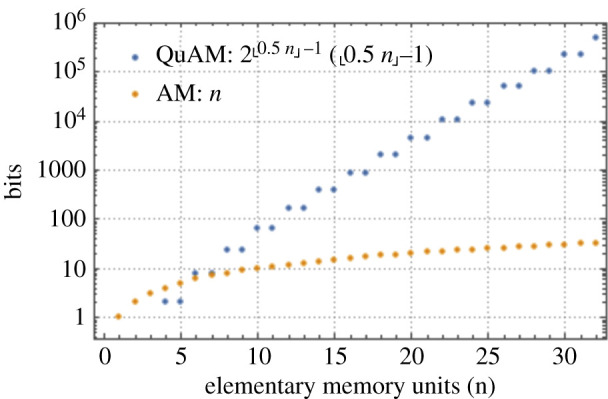
The capacity as a function of the number of elementary physical storage units of quantum and classical associative memories, QuAM and AM, respectively. The elementary storage units are conventional memory cells for classical memory, and 2-level qubits for quantum associative memory. From [36]. (Online version in colour.)

Using quantum associative memory (QuAM) [35] instead of classical associative exploits the potential of quantum computers to provide exponential storage capabilities. Figure 6 shows the number of available bits of information on a logarithmic scale as a function of the number of elementary memory units for classical and quantum associative memory. The storage capacity of the quantum associative memory exceeds that of the classical associative memory once more than eight bits are available and continues to increase exponentially as the number of memory units increases.

**Figure 7.**
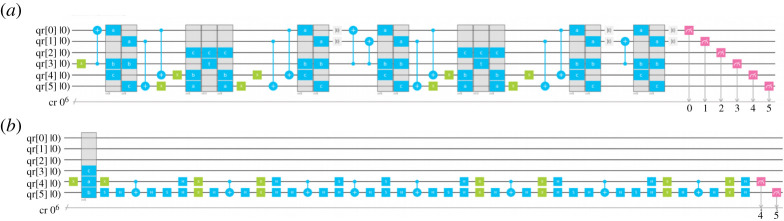
The storage (*a*) and retrieval (*b*) circuits for 2-bit patterns generated by Qiskit. From [36]. (Online version in colour.)

Shapoval & Calafiura [36] present a software implementation of a QuAM protocol for circuit-based quantum computers and demonstrate the quantum circuits needed to store and retrieve a 2-bit pattern. It uses Trugenberger’s algorithm for storage and the generalized Grover’s algorithm for retrieval. The Qiskit framework is used for development. The storage and retrieval circuits for 2-bit patterns obtained for a prototype implementation of QuAM are shown in figure 7. The upper circuit stores three 2-bit patterns and the lower circuit is retrieves a single 2-bit pattern.

## 4. Pattern recognition with quantum graph neural networks

Graph neural networks are a popular technique from machine learning that are currently being explored for a wide range of high-energy physics applications including track pattern recognition [37]. Reference [38] explores how this algorithm could be extended to become a quantum graph neural network, which would run on a circuit-based quantum computer.

**Figure 8.**
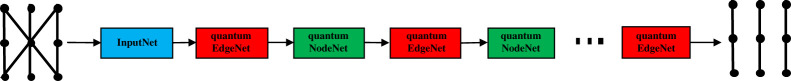
The architecture of the quantum graph neural network. From [38]. (Online version in colour.)

Figure 8 shows the proposed architecture. The hits from the energy deposited in the detector are grouped together into a graph. The input layer is a single layer neural network and increases the dimensions of the input data. Next, the quantum edge network is applied to edge of the graph to calculate edge features which are passed back to the edges. The quantum node network is then applied to each of the nodes of the graph and used to update the nodes in the hidden layers. The edge network and the node network are iterated a number of times until a final quantum edge network is applied to obtain the final segment classification.

**Figure 9.**
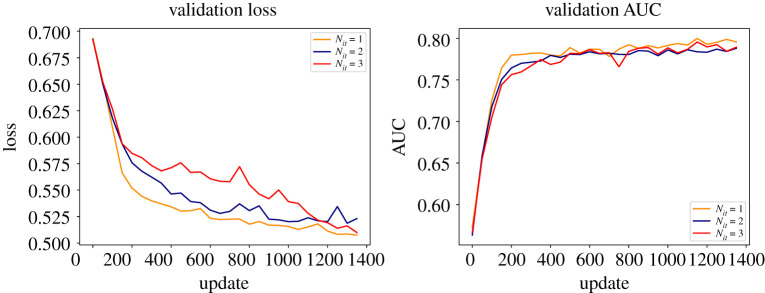
The validation results for a single epoch of training of a quantum graph neural network with different iteration values. From [38]. (Online version in colour.)

The performance of the quantum graph neural network is summarized in figure 9. The validation loss function and the area under the curve are shown as a function of the number of updates for one to three iterations of training the model (*a* and *b*, respectively). While good performance is shown for a single iteration, the performance does not increase with additional iterations, as could be expected.

## 5. Conclusion

The pattern recognition of charged particle trajectories is a particularly challenging computational problem motivating the exploration of novel algorithms. This article has summarized studies of the potential of quantum computers to perform charged particle pattern recognition. A range of approaches were presented targeting both circuit-based quantum computers and quantum annealers. Several implementations of track segment classification algorithms on quantum computers were presented and promising physics performance was demonstrated at expected multiplicities for the HL-LHC. However, the compute performance on the Fujitsu digital annealer was shown to be superior to current quantum implementations. A circuit-based approach to pattern recognition using quantum associative memory was presented. Finally, studies of the use of quantum graph neural networks for charged particle pattern recognition were shown.

Given the current performance of quantum computers, it is currently too early to draw conclusions about which approach is most likely to be successful, but it has been shown that charged particle pattern recognition can be performed on quantum computers.

## Data accessibility

This article has no additional data.
